# Experimental evaluation of low-displacement compression ignited engines operating with hydroxy gas as a supplementary gaseous fuel

**DOI:** 10.1016/j.heliyon.2022.e11545

**Published:** 2022-11-09

**Authors:** Natalia Duarte, Donovan Arango, Geanette Polanco, Guillermo Valencia, Jorge Duarte-Forero

**Affiliations:** a KAI Research Unit, Department of Mechanical Engineering, Universidad del Atlántico, Carrera 30 Número 8–49, Puerto Colombia, Barranquilla, Colombia; b Industrial Technology Department, UiT, The Arctic University of Norway, Narvik, Norway

**Keywords:** Alternative fuels, Diesel combustion, Emissions, Electrolyzer, Hydroxy

## Abstract

A large proportion of annual production of worldwide greenhouses gases results from the use of internal combustion engines. This experimental work evaluates the influence of dual-fuel operation on the overall emissions of a low-displacement compression-ignition engine based on operational conditions such as torque, rotational speed, and load. Hydroxy gas is used as a supplementary gaseous fuel while using pure diespel or palm oil biodiesel as baseline fuels. The CO, CO_2_, NO_x_, and HC emissions levels were carefully characterized through experimental measurements and statistical analysis. The influence of hydroxy enrichment was also examined on the engine's fuel consumption. The study incorporates an in-house hydroxy generator to store and supply the gas in the intake air system using an electrolyzer. The results demonstrated that the ANOVA analysis provides accurate predictions compared to experimental measurements with less than 5% relative error. The use of hydroxy reduces the SFC by up to 25%, which represents an economic advantage of dual-fuel operation, additionally it decreases CO, HC, and CO_2_ emissions. However, with hydroxy enrichment, NO_x_ emissions levels escalate at medium and high loads. Overall, hydroxy enrichment demonstrates to be a robust alternative from an environmental and economic perspective. Future research will be focused on evaluating the biodiesel – hydroxy dual operation, broadening the spectrum of biodiesel concentration percentages, and selecting different raw materials for biofuel production.

## 1 Introduction

Internal Combustion Engines (ICE) are widely implemented for land, sea, and air transportation, electric power generation, and industry. The central role of ICEs in various applications is mainly attributed to their high efficiency, stability, and flexibility for different operating conditions [1, 2]. However, the extensive implementation of ICEs has produced an overwhelming consumption of fossil fuels and alarming rates of greenhouse emissions, specifically, the amount of exhaust gases that are expelled into the environment with contaminants such as CO, HC, SO_x_, NO_x_, PM, and soot [3, 4]. Nowadays, severe regulations have been set by governmental and international entities since these substances alter the balance and the natural composition of air, which represents an imminent risk for human health [5]. Moreover, exhaust gases emanate non-toxic gases such as CO_2_, which accumulate in the atmosphere creating a greenhouse effect that directly contributes to global warming [6]. According to the International Energy Agency (IEA), the energy sector will increase CO_2_ emissions by 50% in 2030 [7]. Therefore, numerous studies have been financed to propose innovative solutions that mitigate emissions levels, especially on thermoelectric plants and vehicles that stand as the largest producers of greenhouse gases [8, 9]. The implementation of biofuels is becoming of increasing interest since it represents a clear solution to reduce fuel consumption and decelerate the depletion of fossil fuels.

According to Bae and Kim [1], alternative fuels are nothing more than those energy sources other than gasoline in the Spark Ignition (SI) engines and diesel in Compression Ignition (CI) engines, which guarantee the integrity of the environment due to reduced emissions and whose source is from a sustainable, renewable and safe energy source. The implementation of biodiesel blends from edible and non-edible feedstocks has been extensively outlined in the literature while displaying promising results toward emissions minimization and enhanced thermal performance [10, 11, 12, 13]. However, there is a significant opportunity for further development and characterization of gaseous fuels incorporation in ICEs, especially in the concrete integration of hydrogen and hydroxy (HHO) gas production systems and subsequent effects on the overall performance and emissions levels [14, 15].

Specifically, hydrogen production is mainly governed by coal gasification and hydrocarbon reforming, accounting for nearly 95% of the global output due to the high-scale and low-cost production [16, 17]. However, the carbon footprint associated with gas reforming technologies significantly affects global warming and fosters greenhouse emissions. Therefore, there is a pressing need for the massive penetration of green-hydrogen systems such as biomass and water-splitting [18]. Notably, the latter has gradually become a promising alternative since water electrolysis enables clean and sustainable production while separating water into its constituent elements, hydrogen, and oxygen. The use of hydroxy in various industrial processes seems an attractive alternative to reducing the use of carbon-based fuels [19, 20, 21].

Certainly, the conceptualization of hydrogen as a reliable fuel that mitigates the harmful effects of fossil fuels in ICEs is supported by its thermophysical characteristics [11, 12, 13]. Accordingly, it is the lightest fuel with the highest energy/weight ratio. The heat released from the reaction of hydrogen, on a mass basis, is about 2.5 times greater than from the combustion of hydrocarbons. Additionally, hydrogen has a wide flammability range, high auto-ignition temperature, low activation energy, high diffusivity, high combustion speed, and a cooling gap three times less than conventional fuels [14, 15]. Hydrogen doping has been successfully implemented in ICEs [22, 23], which are powered by an electrolyzer component. In the same way, hydroxy can be produced via electrolysis in a safe, clean, and renewable way, which outlines a clever option to expand the applications of this gaseous fuel in ICEs.

Recent research has documented the performance of dual-operation in ICEs, with hydroxy gas as s supplemental fuel. Aydin et al. [24] performed an experimental assessment on a diesel engine to reveal the influence of hydroxy gas implementation. The study stated that at speeds below 1750 RPM, the continuous flow of hydroxy decreased the torque delivered by the engine and increased CO, HC, and SFC levels. Therefore, they implemented a Hydroxy Electronic Control Unit (HECU) to decrease the flow of hydroxy at low speed, thus obtaining an average torque increase of 19.1%, a reduction of CO and HC emissions, on average of 13.5% and 5%, respectively, and a decrease in SFC by 14%. In the same vein, Musmar and Al-Rousan [25] reduced NO and NO_x_ emissions by 50%, CO emissions by 20%, and fuel consumption between 20% to 30% in a Honda G 200 engine (single cylinder 197 cc) using a hydroxy generation system with electrolytic cells. Talibi et al. [26] conducted experimental research on a naturally aspirated direct injection diesel engine. The results showed a slight increase in CO_2_ emissions and a significant NO_x_ emissions, with the addition of hydrogen, although only when the temperature exceeded the limit for NO_x_ formation. Likewise, Masjuki et al. [27] mixed hydroxy with ordinary diesel (OD) fuel and 20% (v/v) palm biodiesel with OD (PB20), encountering a 20% and 10% reduction in CO and HC emissions, respectively.

On the other hand, hydroxy generation systems have been implemented on-board in automotive vehicles to perform an in-line replacement. Particularly, Rimkus et al. [28] integrated an electric generator into a vehicle’s engine to produce hydroxy via electrolysis. In this study, hydroxy was incorporated at low concentration levels (0.14–0.18% by volume) in the air. The experimental and numerical results of the study concluded that hydroxy gas ignites at the end of the compression stroke, due to the high pressure and temperature, just before the injection of the diesel fuel. As a consequence of the early ignition of hydrogen, the performance of the engine is drastically affected, presenting an increase in the concentration of CO_2_ and NO_x_ in the exhaust gases. In contrast, they reduced the concentration of incomplete combustion products, such as CO and HC.

So far, extensive assessments have been performed for biodiesel implementation while demonstrating notable improvements in the engine operation that support the utilization of this technique [10, 11, 12, 13]. In terms of gaseous fuel integration, some studies have paved significant appreciations on the overall performance of emissions and fuel metrics. Most of the applications are inclined to perform hydrogen stand-alone gas fuel operation, while the conceptualization of hydroxy gas gradually increases in experimental assessments. Specifically, based on the literature review, there are some contradictions in the characterization of emissions levels behavior since some pollutants increase and others decrease by variating operational parameters such as engine speed, but a concrete qualitative or numerical explanation is rarely found. The latter can be carefully clarified by analyzing the influence of operational parameters in a multivariate context that further unravels performance patterns that facilitate accurate dual-fuel operation prediction. Moreover, the integration of numerical and statistical models stand as robust tools to define the global dual-fuel operation pattern.

The main objective of this investigation is to determine the influence of hydroxy gas as a supplementary fuel on the mechanical, thermal; environmental such as the potential impact on improving emissions and the benefits that can be obtained from being implemented in a low-displacement CI engine; and the effect this represents on an economically. Accordingly, an in-house test bench is designed while comprising a stationary engine and an electrolytic cell with a solution of KOH-distilled water for hydroxy production. Moreover, a Data Acquisition (DAQ) system is integrated to record the output variables of the test bench and two gas analyzers to quantify the emissions levels. The study evaluates the impact of operational parameters such as engine load, engine speed, and volumetric gas flow addition. Notice that the experimental assessment alternates between pure diesel (PD) and palm oil biodiesel (B10) as baseline fuels. The incorporation of a complete statistical model based on ANOVA analysis to characterize the overall performance in a multivariate context represents a remarkable aspect of this investigation. Therefore, this work facilitates closing the knowledge gap immersed in dual-fuel operation in diesel engines and green-hydrogen technologies integration. The paper is structured as follows: Section 2 describes the main aspects of the experimental assessment and describes the characteristics of the dual-fuel operation. Later, Section 3 presents the main outcomes of the investigations while outlining critical discussions. Finally, Section 4 establishes the concluding remarks, limitations, and future avenues in the field.

## 2 Materials and methods

### 2.1 Experimental setup

**Figure 1:**
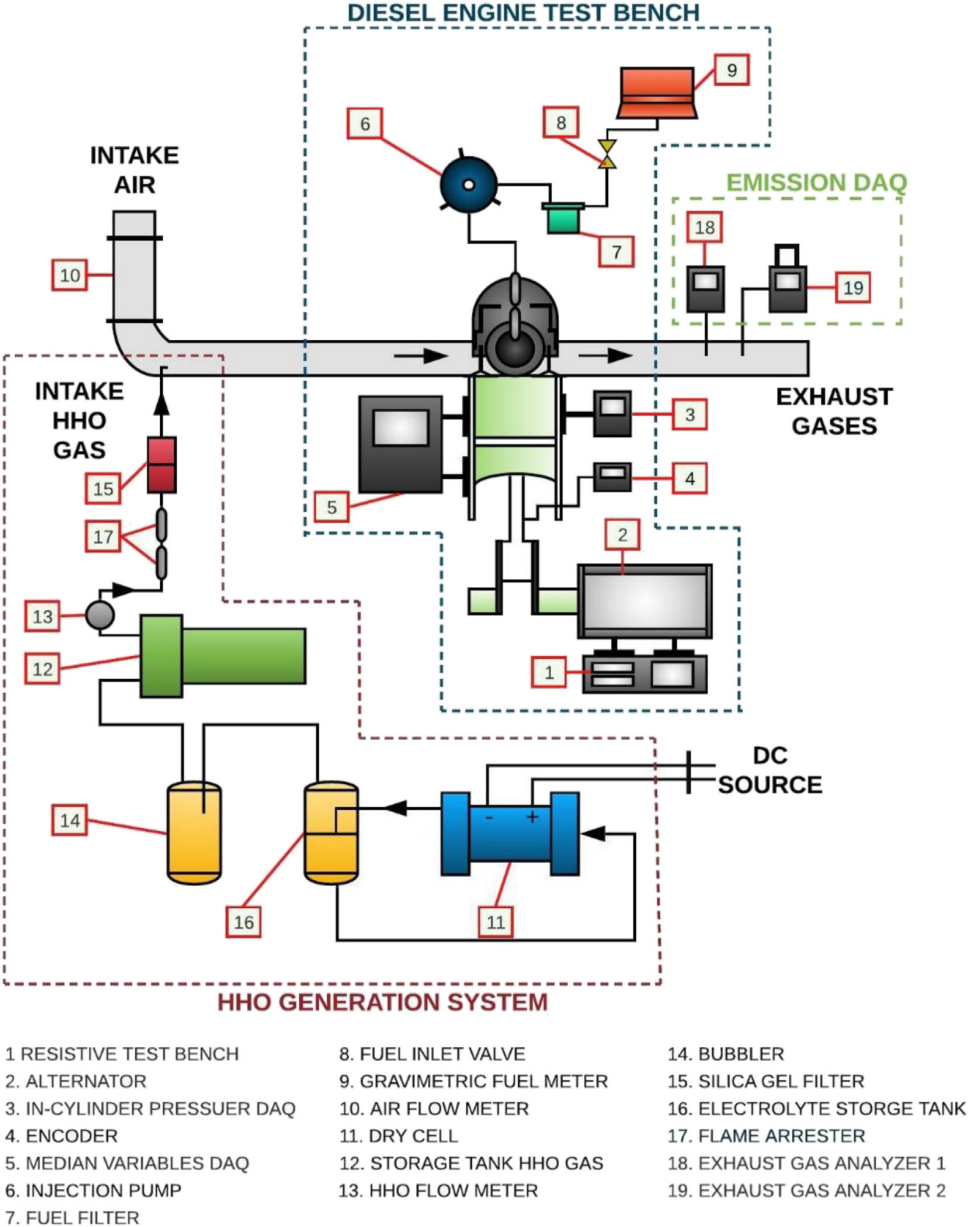
Schematic representation and configuration of the equipment.

Figure 1 schematically depicts the arrangement of the experimental test bench used in the study. Overall, three main groups in the setup can be identified: diesel engine test bench, HHO generation system, and Emission Data Acquisition (DAQ) system.

The diesel engine test bench consists of a SOKAN SK-MDF300 engine, which is a single-cylinder, low-displacement, four-stroke internal combustion engine. It also has a naturally aspirated intake system and a direct injection system with a Before Top-Dead Center (BTDC) angle of 20°. The cylinder bore and stroke are 78 mm and 63.57 mm, respectively, enabling a displaced volume of 299 cc and a compression ratio of 20:1. The power delivered by the engine is controlled by the resistive test bench (1), and it reaches a maximum value of 4.6 hp at 3600 rpm. A set of sensors and indicators are part of the DAQ system (5) that serves to visualize and record pressures and temperatures at key points of the engine. For the fuel supply, there is a system that consists of an injection pump (6), fuel filter (7), and valve (8). The bench also has a gravimetric meter (9) to quantify fuel consumption and a Mass Air Flow (MAF) sensor (10) to measure the air in the intake engine.

The HHO generation system, shown in Figure 1, is designed to produce and store the hydroxy produced by the electrolyzer. To increase or decrease the rate of production of hydroxy, a Pulse Width Modulator (PWM) varies the average voltage at which the cell (11) is fed by varying the duty cycles with the potentiometer located on the instrument panel in the section of electrical parameters. In this section, an ammeter shows the intensity of the current flowing through the electrolytic cell, and a voltage meter the voltage of the source that feeds the PWM. Lastly, a manometer located in one of the hydroxy tanks (12) allows for recording the pressure variation and estimating the production rate. Moreover, the partial fuel replacement to the CI engine is measured using a flow meter (13) installed at the exit of the hydroxy tank. To minimize the presence of humidity in the HHO gas generated, a bubbler (14) is located before the hydroxy tank, and a silica gel filter (15) is located downstream from it. The tank with electrolyte (16) that was designed and implemented in this experiment is 316L stainless steel. For safety, to prevent flashbacks, a flame arrester (17) was installed in the connection line between the engine and the storage tanks.

The emission DAQ section consists of two gas analyzers. The first gas analyzer installed in the exhaust line corresponds to a Bacharach® model PCA 400 portable (18) with O_2_, CO, NO, NO_2_, and SO_2_ sensors, and the second gas analyzer is a Brain Bee AGS-688 (19) with an HC sensor. The measuring instrument of O_2_ has a range of 0–21% v/v and accuracy of ±0.3%. For CO and NO, the range is 0–10000 ppm, and the accuracy is ±10%. The NO_2_ has a range of 0–1000 ppm and an accuracy of ±5%. Finally, the range and accuracy for the measurement of HC are 0–19999 ppm and ±1%, respectively.

### 2.2 Experiment design

**Figure 2:**
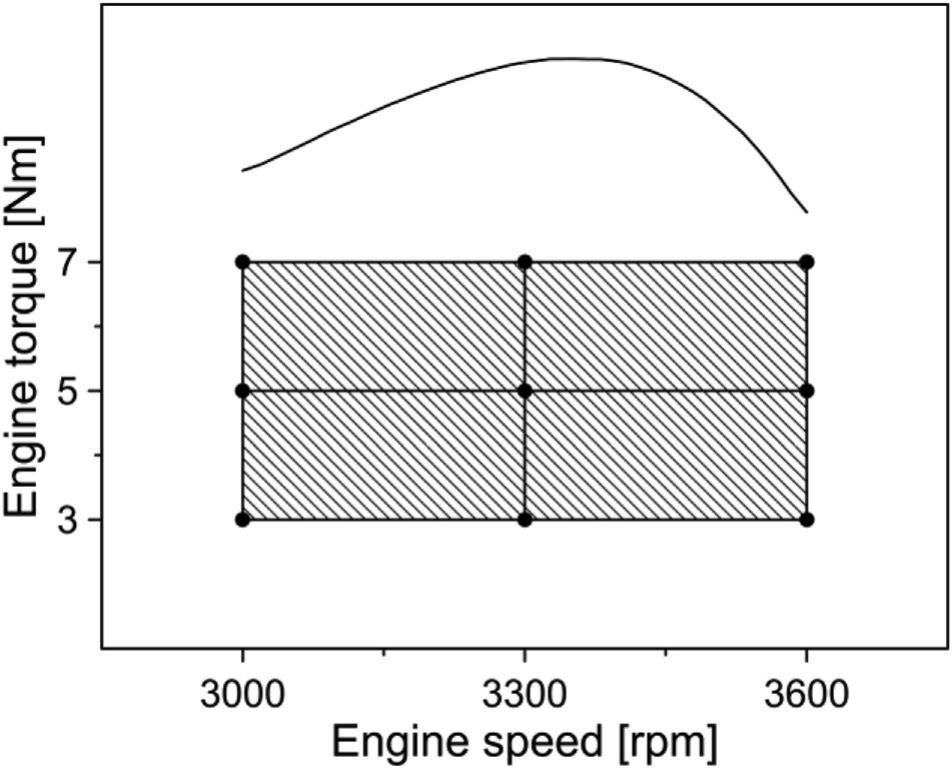
Engine operating conditions.

This section aims to describe the main characteristics of the experimental assessment. The operating modes selected in the experiments were established, taking into account the engine power curve, the capacity of the resistive test bench, and the operating conditions of the power bench. Then, the operating ranges for torque and speed were set from 3 to 7 Nm and from 3000 to 3600 RPM, respectively. Three load conditions, namely low-load (3 Nm), medium-load (5 Nm), and high-load (7 Nm), were established to perform the experiments. The load and rotational speed conditions were selected to cover the entire operating area to which the engine can be subjected. In this way, it is possible to evaluate the performance and emissions in the different operating conditions of the engine, as shown in Figure 2.

Regarding partial fuel substitution with hydroxy gas, the production and storage capacity of the electrolyzer is defined as the maximum allowable flow, which corresponds to 8 LPM. For comparison schemes, a baseline case of 0 LPM was incorporated into the analysis. Moreover, the tests were carried out alternating between pure diesel and palm biodiesel at 10% replacement (90% diesel +10% palm biodiesel) as the main fuels. The B10 biofuel was chosen because this substitution percentage does not cause a significant change in the properties of pure diesel. Additionally, B10 is the regulated biodiesel in the Colombian context. Table 1 lists a matrix of the fuel properties considered in the experiment.

**Table 1:** Main input properties of pure diesel, biodiesel, and gaseous fuels.

Property	Standard	PD	B10	H_2_	HHO
Average molecular weight [kg/kmol]	-	170	-	2.01594	12.01
Density [kg m^−3^]	ASTM D1298	821.5	827.5	0.08376	0.49115
Viscosity [cSt]	ASTM D445	2.64	2.66	-	-
Flashpoint [°C]	ASTM D93	76	96	-	-
Cloud point [°C]	ASTM D2500	6.5	8.3	-	-
Pour point [°C]	ASTM D97	3.1	3.8	-	-
High heating value [MJ kg^−1^]	ASTM D2015	44.05	43.25	142.18	25.982
Low heating value [MJ kg^−1^]		42.5	-	120.21	21.995
AIT [°C]	-	257	-	536–585	-
Stoichiometric air/fuel ratio [mass basis]	-	14.5	-	34.12	-
Flamability limit [% volume]	-	0.6–5.5	4.0–74.5		

### 2.3 Uncertainty analysis

For the analysis of the uncertainty of the experimental measurements, the Type A evaluation method was used, which consists of statistical analysis through a series of measurements. To determine the best estimate of a series of measures, Eq. (1) was used.

(1) $$\bar{x}=\frac{\sum_{i=1}^{n}x_{n}}{n}$$

where n represents the number of measurements. To determine the dispersion of the series of measures, the standard deviation (s) was used, as shown in Eq. (2).

(2) $$s=\sqrt{\frac{\sum_{i=1}^{n}{\left(x_{n}-\bar{x}\right)}^{2}}{n-1}}$$

Finally, the uncertainty (u) of the series of measurements was calculated using Eq. (3).

(3) $$u=\frac{s}{\sqrt{n}}$$

In the case of parameters subject to more than one source of uncertainty, the calculation of a combined uncertainty $\left(u_{c}\right)$ was performed, as shown in Eq. (4).

(4) $$u_{c}=\sqrt{{\left(u_{1}\right)}^{2}+{\left(u_{2}\right)}^{2}+{\left(u_{3}\right)}^{2}+\ldots +{\left(u_{n}\right)}^{2}}$$

## 3 Results and discussion

### 3.1 Sensitivity analysis

#### 3.1.1 Specific fuel consumption (SFC)

**Figure 3:**
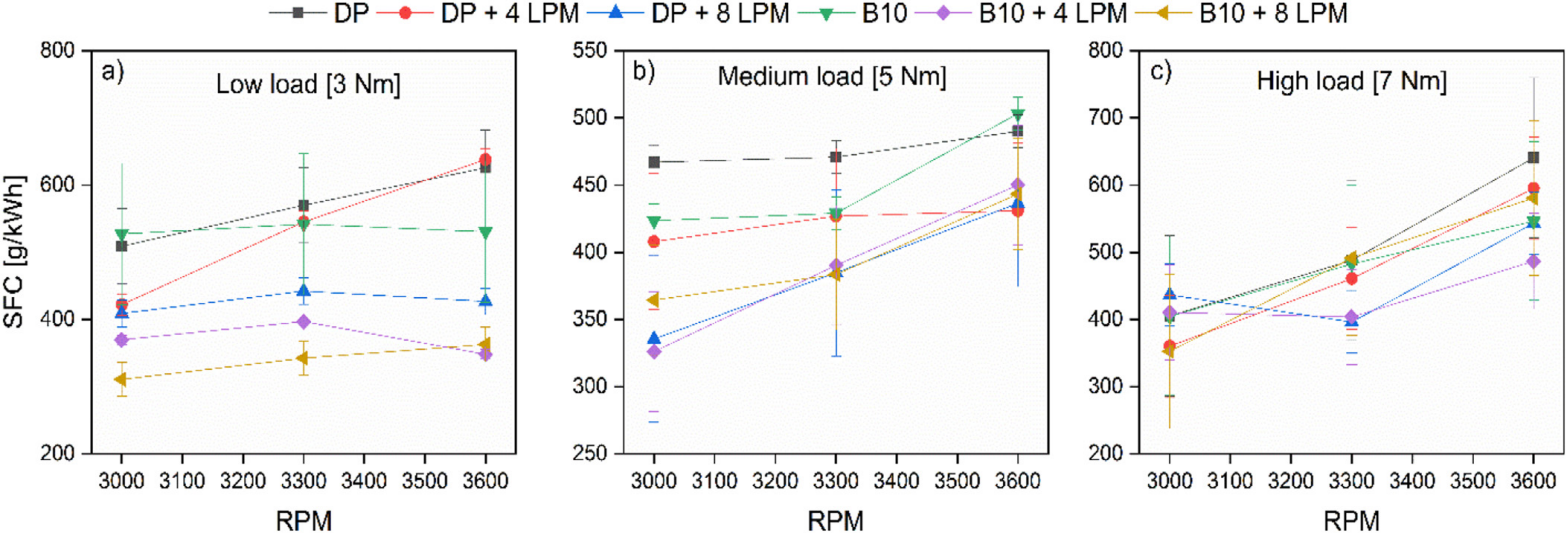
Specific Fuel Consumption (SFC) at (a) low, (b) medium, and (c) high loads.

Figure 3 displays the influence of the tested fuel, engine load, and engine speed on the SFC behavior.

According to the results of Figure 3, it can be noticed that the overall trend of the SFC is not quite intuitive. In some cases, the B10-hydroxy operation features reduced SFC. However, in other scenarios, the hydroxy-PD provides the lowest share. To a great part of the results, enriching the intake air with hydroxy promotes SFC reduction in both PD and B10, although this behavior differs with increasing the RPM. For instance, at minimal load, the best performance is achieved by incorporating biodiesel blends and maximum hydroxy flows (8 LPM), except the case at maximum engine speed, in which 4 LPM enables the lowest fuel consumption. Interestingly, at minimal load and low speed, the difference between increasing the HHO from 4 to 8 LPM is not significant when operating with pure diesel. However, when the RPM rises, the 4 LPM case boosts the SFC, while the 8 LPM operation promotes a significant reduction.

Regarding the B10 curves, the stand-alone B10 operation presents the greatest fuel consumption at a low-speed regime at low load. However, as the RPMs rise, the PD operation features greater SFC values. At medium load, the SFC decreases for PD and B10 due to the efficiency of the combustion process. In this case, like the previous one, the flow of hydroxy tends to decrease the response variable. However, as it can be observed, at medium load, increasing the flow of hydroxy from 4 to 8 LPM of hydroxy does not have a strong incidence on the SFC with B10 since the same result is obtained for PD + 8 LPM of hydroxy and B10 + hydroxy. The latter represents a remarkable aspect since reducing SFC only with hydroxy incorporation is possible, which represents an economic advantage as the biofuel can be eliminated. At maximum load, the effect of PD + hydroxy is the same as at low load. In contrast, a mixture of B10 + 4 LPM of hydroxy shows better results than a flow of 8 LPM of hydroxy.

For B10, the SFC is reduced by 5.3% and 22%, both for B10 + 4 LPM and B10 + 8 LPM for hydroxy. The quantity of hydroxy is a variable with a strong incidence on the quality of the combustion process of PD and B10, also taking into account the operating condition. At a low load, a high flow of hydroxy interferes with oxygen intake and causes inefficient combustion [24]. On average, the use of hydroxy as a supplementary fuel decreases fuel consumption, which agrees with the results reported by Ismail et al. [29].

#### 3.1.2 CO_2_ emissions

**Figure 4:**
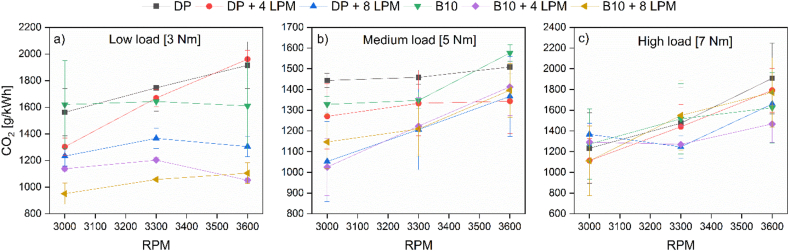
CO_2_ emissions at (a) low, (b) medium, and (c) high loads.

Figure 4 shows the overall trend of carbon dioxide emissions for the different tested fuels and hydroxy gas replacement.

The overall trend of Figure 4 shows that CO_2_ emissions are directly related to the amount of liquid fuel used in this experiment, which explains why the curves resemble those displayed for the SFC. Decreasing the SFC by enriching the intake air with hydroxy, on average, decreases the concentration of CO_2_ in the exhaust gases by 20%. This trend is in agreement with Thangaraj and Govindan [30]. The reduction of this carbon dioxide is derived from factors such as combustion temperature and the C/H ratio [31]. Additionally, the absence of carbon molecules in hydroxy gas hinders the formation of CO_2_ and CO during the combustion process [32]. Specifically, the CO_2_ emissions for the PD operation remain at the highest levels at low and medium engine load, expecting at a maximum engine speed where the pollutants levels are overpassed by the PD + 4LPM for the former and B10 for the latter. On the other hand, the optimal operation for emissions minimization fluctuates at a high engine load since the PD stand-alone operation features a lower share at low and medium engine speed than the B10 and B10 + 8 LPM. Interestingly, the emissions differential is significantly higher at low engine loads, whereas the emissions differential is reduced at high engine loads. The latter indicates that the emissions levels of CO_2_ are directly related to the temperature gradient developed during combustion, which is significantly higher at high loads and subsequently produces greater emissions; thus, the positive effects of the dual-fuel operation are not substantial as in the low-load scenario.

#### 3.1.3 CO emissions

**Figure 5:**
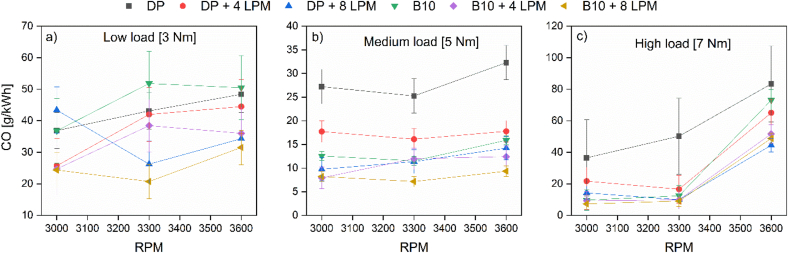
CO emissions at (a) low, (b) medium, and (c) high loads.

The CO emission levels of the experimental assessment are reported in Figure 5. When analyzing the curves shown in Figure 5, it is observed that CO emissions do not vary significantly with increasing RPM for PD operation at low and medium loads. The drastic changes in the response variable are evident at high load since, in this case, the A/F ratio is below the stoichiometric value. Therefore, the combustion process is very inefficient. By replacing PD fuel with hydroxy at low load and low speed, a flow of 8 LPM of hydroxy negatively affects the combustion process, increasing the concentration of CO in the exhaust gases. Keeping the engine at low load at medium and high engine speed, a hydroxy volumetric flow of 8 LPM reduces emissions of this pollutant by an average of 34%, while a 4 LPM flow of hydroxy, by only 5%.

On the other hand, the B10 stand-alone operation increases CO emissions by an average of 8% when the engine operates at a low load. However, increasing hydroxy flow (4–8 LPM) changes this condition by reducing CO emissions by an average of 40%.

As mentioned above, the combustion process is more efficient at a medium load through the engine speed margin. In consequence, CO levels are stable, and its concentration in the exhaust gases decreases. For the B10 scenario, the CO emissions are reduced up to 53% at medium load. Moreover, incorporating hydroxy gas further reduces the pollutant concentration by 58.4% for the PD + 8 LPM of hydroxy and a maximum of 70.8% for B10 + 8 LPM of hydroxy. This behavior is maintained even when increasing the load to 7 Nm (high load) with low and medium engine speed.

Specifically, at high load, the sudden peak of CO levels is attributed to the high fuel demand that the engine requires to reach maximum power according to the fashion of the performance curve. However, when the intake air is enriched with a hydroxy flow of 8 LPM, CO emissions are reduced by 42% for B10 and 46.7% for PD. Moreover, CO emissions are reduced by 31.8% for PD + 4 LPM and 48.1% with 8 LPM of hydroxy flow. When the engine works only with B10, CO emissions are reduced by 32.8%. For B10 + 4 LPM hydroxy blends, this emission is reduced by 49.7% and up to 59.6% when the hydroxy flow increases to 8 LPM. These results are compatible with findings reported by Sharma et al. [33]. In general, it was observed that the presence of hydroxy gas favors the reduction of CO emissions, which may be associated with the diffusivity of this gaseous fuel and the better air/fuel mixture. The above factors contribute to a more homogeneous mixture and a shorter duration of the combustion process [34].

#### 3.1.4 NO_x_ emissions

**Figure 6:**
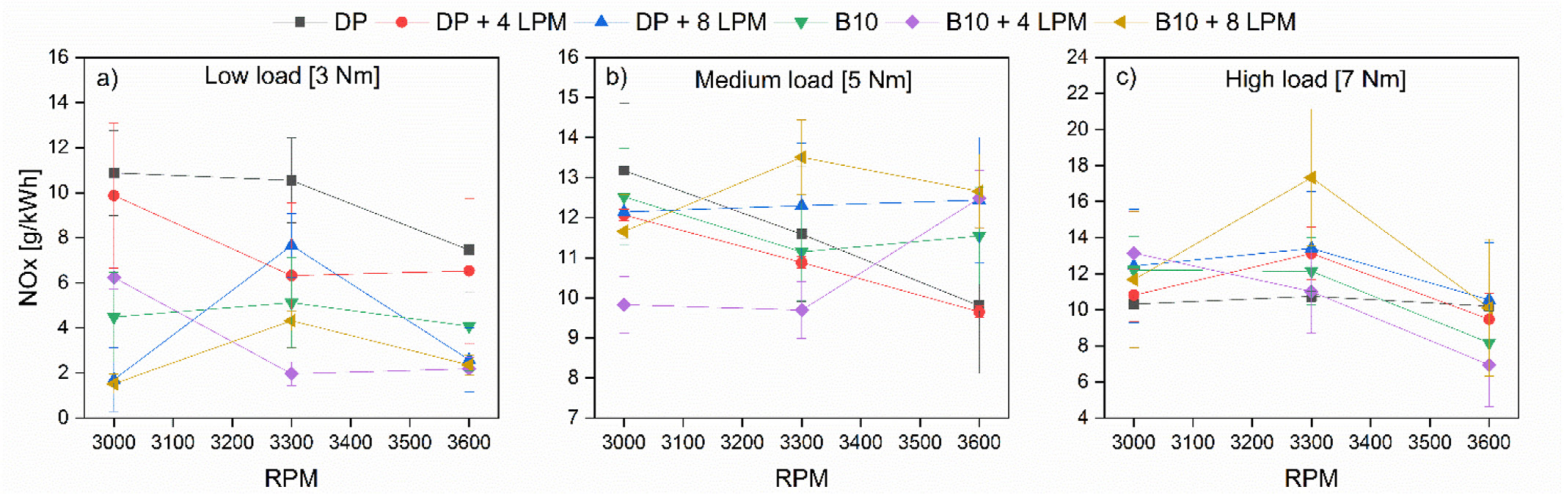
NO_x_ emissions at (a) low, (b) medium, and (c) high loads.

Figure 6 displays the overall trend of the NO_x_ emissions for the tested fuels and the engine's operating range.

According to the results shown in Figure 6, dual-fuel operation with biodiesel blends and hydroxy promotes NO_x_ emissions, expecting low loads. Also, at the highest volumetric flow enrichment (8 LPM), the pollutant emissions significantly rise at a medium speed margin for both the PD and B10 operation. In contrast, a medium replacement level guarantees NO_x_ minimization. It is worth mentioning that the A/F ratio in CI engines, over a wide operating range, is below the stoichiometric value. This represents an excess of air in the combustion process, favoring the formation of NO_x_ [35]. This is why the emissions of this pollutant for PD, as shown in Figure 6, remain at a high level regardless of the load imposed on the engine and with little variation according to the RPM. In addition to excess air, temperature is a key factor in the different mechanisms of NO_x_ production. Consequently, the low-load B10 mixture lowers the temperature of the combustion process, thus reducing NO_x_ formation by an average of 51%. Hydroxy follows this trend at low load but with strong interactions depending on flow and RPM: at low speed, PD + 8 LPM of hydroxy reduces emissions by 84%; at an intermediate speed, B10 + 4 LPM reduces emissions by 81%; And at a high rate, B10 + 4 LPM reduces emissions by 70%. The combustion chamber temperature increased dramatically for each investigated mixture as the load increased. At medium load and low speed, the NO_x_ levels resulting from the different fuel mixtures are below the values registered for PD, although only B10 + 4 LPM presents a notable reduction of 25%. In these three modes of operation at medium load, the PD + 4 LPM mixture reduces the emissions of the variable response by an average of 5%. At the same time, a flow of 8 LPM plus PD or B10 increases the emissions by an average of 16 % and 23%, respectively, at intermediate and high RPM. The tendency to decrease NO_x_ emissions at low and medium load regimes differs at maximum load. In this case, at low RPM, PD + 4 LPM of hydroxy increased NO_x_ emissions by 4.8%, but by increasing the flow to 8 LPM with PD, NO_x_ emissions increased an average of 27% for a low-intermediate speed margin. The latter indicates a considerable increase in the temperature of the combustion process by enriching the intake air with hydroxy when the engine works at a high load.

On the other hand, a mixture of B10 + 4 LPM of hydroxy gas at intermediate speed shows a slight increase (2%) in the emissions levels compared to the PD stand-alone operation. In the operation mode with the higher power, high speed, and high load, this mixture (B10 + 4 LPM) reduces emissions by 32%. This result can be explained considering that the A/F ratio is close to the stoichiometric value, reducing excess air despite the high temperature. This pattern is in line with the results reported by related investigations [22, 24].

#### 3.1.5 HC emissions

**Figure 7:**
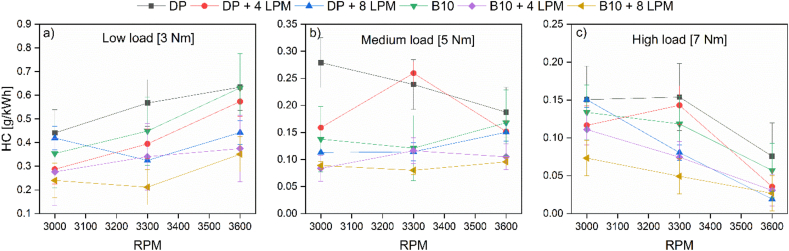
HC emissions at (a) low, (b) medium, and (c) high loads.

This section concludes with the characterization of HC emissions levels as reported in Figure 7.

According to the results, the dual-fuel operation mode presents clear benefits toward HC minimization since the PD stand-alone operation features the highest pollutant levels except at medium load and medium engine speed. The speed of rotation and the load imposed on the engine has a strong incidence on HC emissions, as shown in Figure 7. At low loads, the concentration of HC increases with the increasing speed of rotation, while at medium and high loads, the opposite effect is observed. The concentration of HC in the exhaust gases depends, among other factors, on the efficiency of the combustion process according to the homogeneity of the fuel mixture and the spread of the flame. Notice that at low load, the highest levels of the SFC and A/F ratio for PD were recorded; therefore, under these conditions, the combustion process has a low efficiency that is reflected in the amount of unburned HC since the zones within the combustion chamber are increased with poor mixtures and/or rich mixture zones. This effect is increased in the vicinity of the cylinder walls by heat transfer that inhibits the appropriate conditions to initiate chemical reactions. By replacing fossil fuel with biodiesel at a 10% share, an average reduction in HC emissions of 14% was recorded. Another contributor to this pattern is the narrow cooling gap of the hydroxy's combustion and the rapid spread of its flame front. In the case of the B10 + 8 LPM mixture, the overall emissions drop by an average of 51%. It should be noted that an 8 LPM flow of hydroxy + PD at low load and low regime reduced HC emissions by only 5%, as the high flow limited the amount of air affecting the combustion process. This trend is supported by the results of the mixture PD + 4 LPM of hydroxy, which enable an emission reduction of 35%.

By enriching the air with hydroxy when the engine works at maximum load and high speed, that is, the maximum power for this experiment, HC emissions decrease to 25% of those emitted when the engine works only with PD, this being the mode of operation with higher fuel consumption per unit of time. The results are in line with the outcomes reported by Yilmaz et al. [24] and Rimkus et al. [28]. The content of oxygen present in the hydroxy gas allows for improving the mixing of the air/fuel. Additionally, the high speed of the hydroxy flame leads to an increase in the combustion temperature, causing the oxidation of hydrocarbons. The foregoing allows explaining the reduction in HC emissions in fuel mixtures with hydroxy [36].

#### 3.1.6 Particulate matter emissions

**Figure 8:**
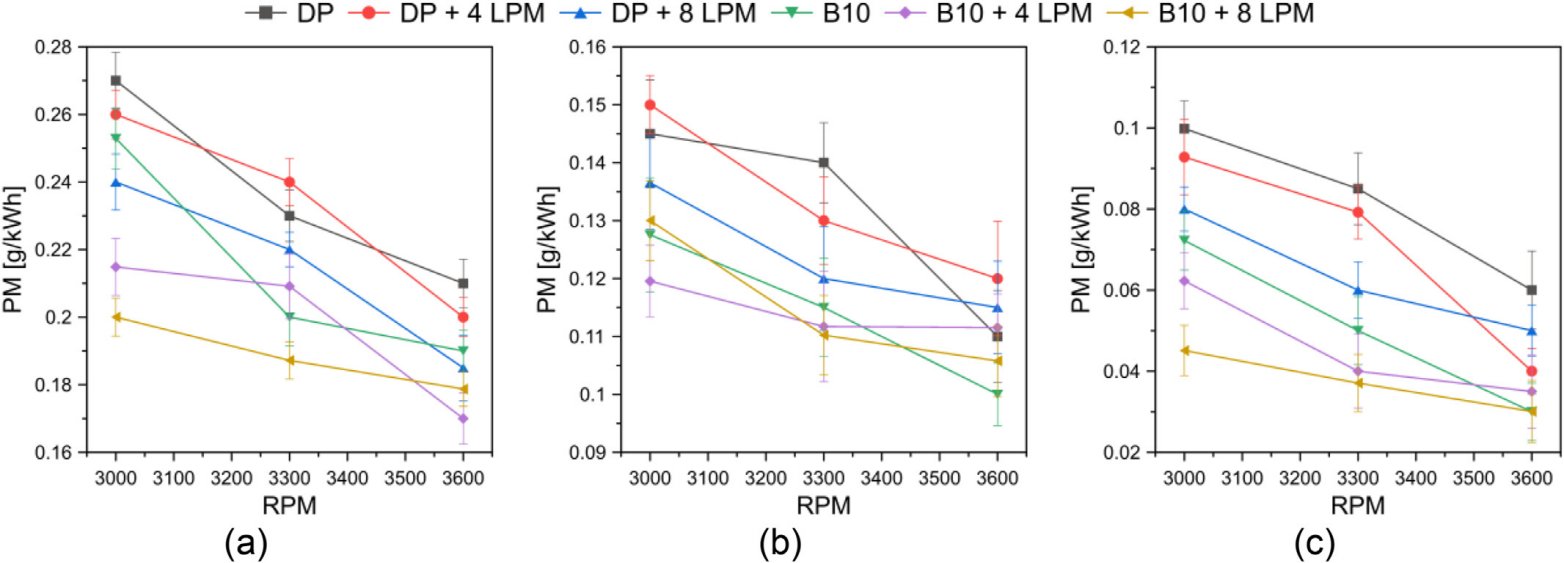
PM emissions at (a) low (3 Nm), (b) medium (5 Nm), and (c) high (7 Nm) loads.

Figure 8 shows the trend of particulate matter (PM) emissions for the fuels tested and the engine's operating range.

In general, particulate matter is produced due to an incomplete combustion process. The results obtained indicate that enrichment with hydroxy gas leads to a decrease in PM emissions. This can be attributed to the absence of carbon in the hydroxy gas. On average, the addition of 4 LPM and 8 LPM volumetric flow caused an 8.9% and 12.1% reduction in PM emissions for conventional diesel. In the case of B10, a reduction of 11.3% and 13.1% was observed with the addition of a volumetric flow of 4 LPM and 8 LPM, respectively.

### 3.2 Regression model

#### 3.2.1 Model development

A compound statistical model was developed considering the relationship between emissions, engine torque, engine speed, and hydroxy flow for the two selected fuel types (B10 and PD) from the experimental results reported in the previous section. Accordingly, the p-value of each term was analyzed by comparing it with a significance level of 0.05 to determine the statistical significance of the relationship between the response variable and the analyzed term. Thus, it was established that for p-values greater than 0.05, the relationship between the term and the response variable is not significant. On the contrary, for p-values smaller than 0.05, the model considers the term since its association with the response variable is significant. Table 2 shows the p-values of each of the terms considered for the development of the models.

**Table 2:** P-values of the ANOVA analysis.

	CO		CO_2_		NO_x_		HC	
Factor	PD	B10	PD	B10	PD	B10	PD	B10
R	0.0000	0.0000	0.0000	0.0000	0.4313	0.7512	0.0001	0.0000
T	0.0000	0.7298	0.0000	0.0000	0.0000	0.0000	0.0000	0.0000
H	0.0000	0.0001	0.0001	0.0459	0.0023	0.0000	0.0000	0.0000
$R^{2}$	0.0000	0.0000	0.0000	0.0000	0.1561	0.1161	0.3833	0.0008
RT	0.0000	0.0000	0.0000	0.0000	0.7334	0.1600	0.0000	0.0248
RH	0.0170	0.3421	0.0050	0.5277	0.0537	0.2167	0.0429	0.0000
$T^{2}$	0.0000	0.0000	0.2288	0.0158	0.0000	0.0000	0.0531	0.8191
TH	0.0000	0.6611	0.1246	0.2552	0.0000	0.0021	0.9600	0.8574
$H^{2}$	0.7915	0.2217	0.0755	0.4779	0.6859	0.2978	0.7950	0.9820

Terms $R^{2}$, RT, RH, $T^{2}$, TH and $H^{2}$ contain the corresponding values for the terms that involves the multiplication of two of the main variables (R: speed, T: torque and H: hydroxy flow), respectively.

**Figure 9:**
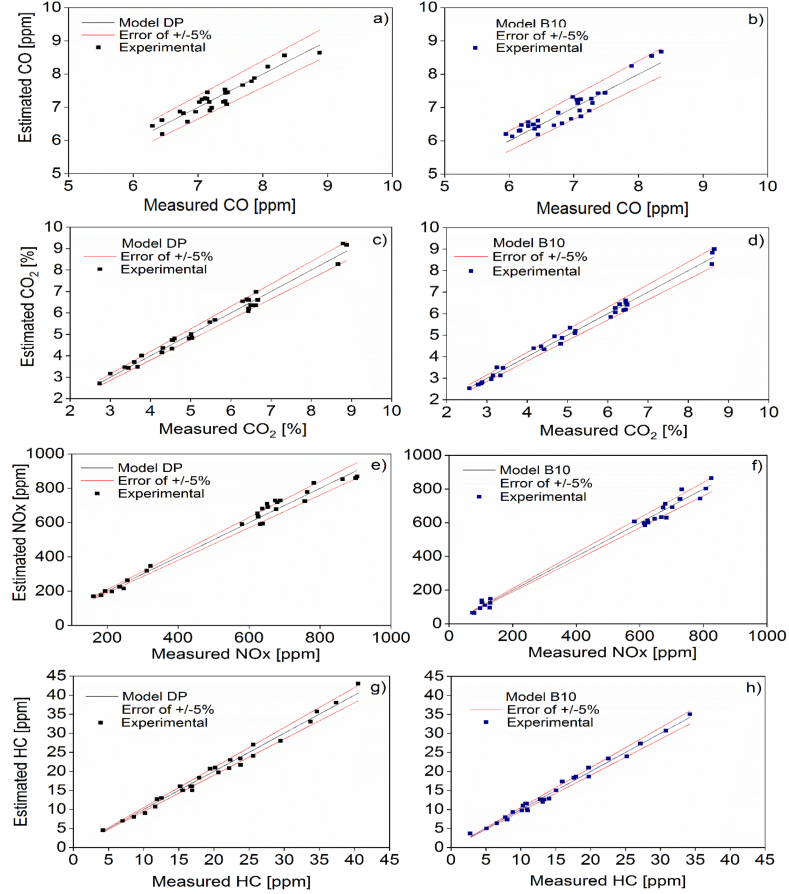
Models’ validation for the statistical model for the emissions of CO in (a) PD and (b) B10 modes; CO_2_ in (c) PD and (d) B10 modes; NO_x_ in (e) PD and (f) B10 modes; HC in (g) PD and (h) B10 modes.

Table 3 shows the regression models obtained for the two baseline fuels. These models were adjusted with 80% of the experimental data through the ANOVA analysis. Likewise, these models were validated considering the remaining 20% of the experimental data, whose behavior are shown in Figure 9. The overall trend of the results indicates that the regression models fit the data studied with an error range of ±5%.

**Table 3:** Statistical models of the emissions levels of the engine.

Emission	Model	
	PD	B10
CO	$\log\left(CO\right)=48.4913-22.8831x10^{-3}R-22.4567x10^{-1}T-18.0804x10^{-2}H+32.8778x10^{-7}R^{2}+40.7890x10^{-5}RT-78.1202x10^{-6}RH+11.4177x10^{-2}T^{2}-34.1319x10^{-3}TH$	$\log\left(CO\right)=57.6444-26.7016x10^{-3}R+13.6091x10^{-2}H+37.7165x10^{-7}R^{2}+71.138x10^{-5}RT+16.9782x10^{-2}T^{2}$
${CO}_{2}$	${CO}_{2}=67.766-38.6088x10^{-3}R-25.3194x10^{-1}T-51.0359x10^{-2}H+{56.4815x10^{-7}R}^{2}+91.8981x10^{-5}RT+14.4676x10^{-5}RH$	${CO}_{2}=64.5557-35.838x10^{-3}R-30.5718x10^{-1}T-13.5012x10^{-2}H+48.5597x10^{-7}R^{2}+13.4722x10^{-4}RT-49.7685x10^{-3}T^{2}$
${NO}_{X}$	${NO}_{X}=-2969.43+41.5771x10^{1}T-10.9997x10^{1}H+18.0185x10^{-3}RH-34.6042T^{2}+10.8965TH$	${NO}_{X}=-4953.97+78.8879x10^{1}T-55.6000H-56.0574T^{2}+47.1181x10^{-1}TH$
HC	$HC=-221.355+10.8704x10^{-2}R+28.912T-42.3958x10^{-1}H-11.2037x10^{-3}RT+94.9074x10^{-5}RH+38.4259x10^{-2}T^{2}$	$HC=117.448-0.0831944∙R+11.5741T+16.9097x10^{-1}H+19.9588x10^{-6}R^{2}-81.0185x10^{-4}RT-69.4444x10^{-5}RH$

R:[RPM],T:[Nm]andH:[lpm]

#### 3.2.2 Response surfaces

The main contribution of this section is to serve as a handy tool to determine the operating conditions that guarantee minimal emissions levels for the different pollutants analyzed. Considering the regression models obtained, the response surfaces were represented for each of the emissions studied to verify the behavior presented in the previous sensitivity analysis. The latter takes relevance considering that the emissions maps displayed are derived from the statistical model in a multivariate context that involves the main operation parameters in the engine.

**Figure 10:**
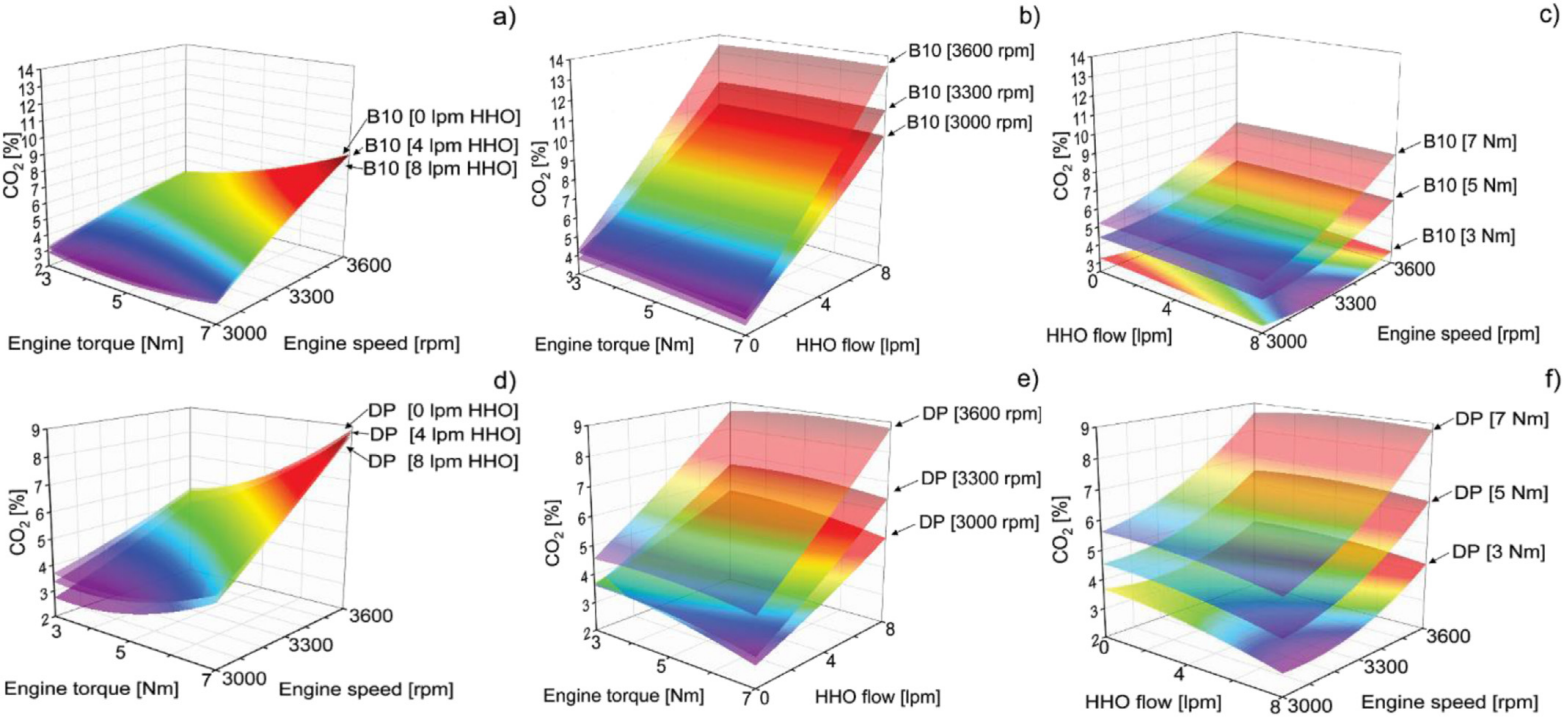
CO_2_ response surfaces of B10 as a function of a) hydrogen volumetric flow b) engine speed, and c) torque and CO_2_ response surfaces of DP as a function of d) hydrogen volumetric flow e) engine speed, and f) torque.

Firstly, in the case of the response surfaces for CO_2_, the results in Figure 10 indicate that there is a direct proportion between the emissions, the engine speed, and the engine torque. Thus, it was found that the minimum values of CO_2_ emissions can be obtained by operating the engine at low engine speed and torque, keeping the flow of hydroxy constant. This pattern is in line with other studies that concluded that CO_2_ emissions directly relate to the engine load while operating at constant engine speed [26, 27, 28]. Regarding the flow of hydroxy, CO_2_ emissions are minimal when the flow of hydroxy is at a maximum volumetric rate.

**Figure 11:**
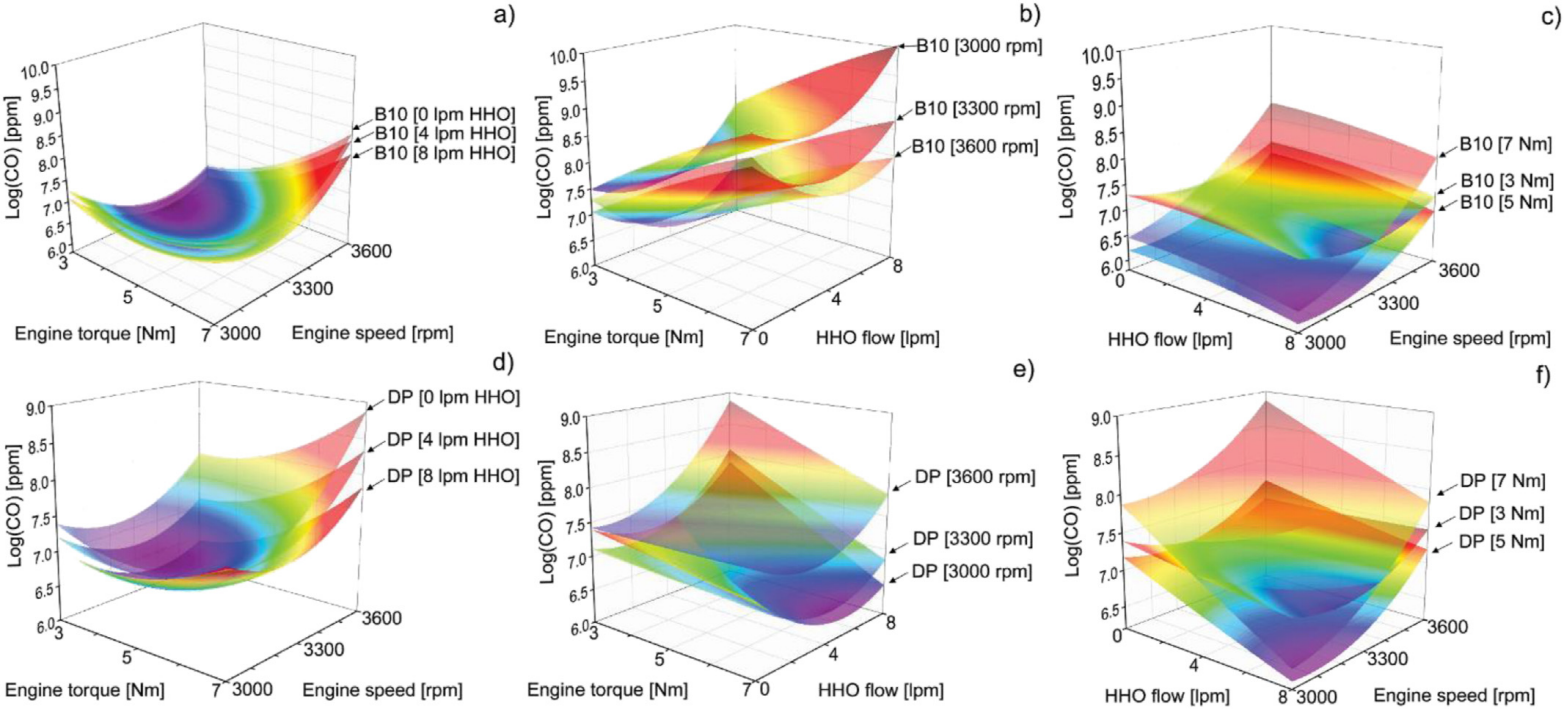
CO response surfaces of B10 as a function of a) hydrogen volumetric flow, b) engine speed and c) torque and CO response surfaces of DP as a function of d) hydrogen volumetric flow, e) engine speed and f) torque.

Subsequently, the response surfaces in Figure 11 indicate that the minimum values for CO emissions are obtained when torque is increased at constant engine speed or if the engine speed is increased at constant torque. These results are consistent with several studies showing a decreasing CO emissions trend when engine torque is increased at a constant engine speed [26, 29, 30]. As for the association between the emission of CO and the flow of hydroxy, it is observed that the values are minimized when the flow of hydroxy increases for both pure diesel and biodiesel.

**Figure 12:**
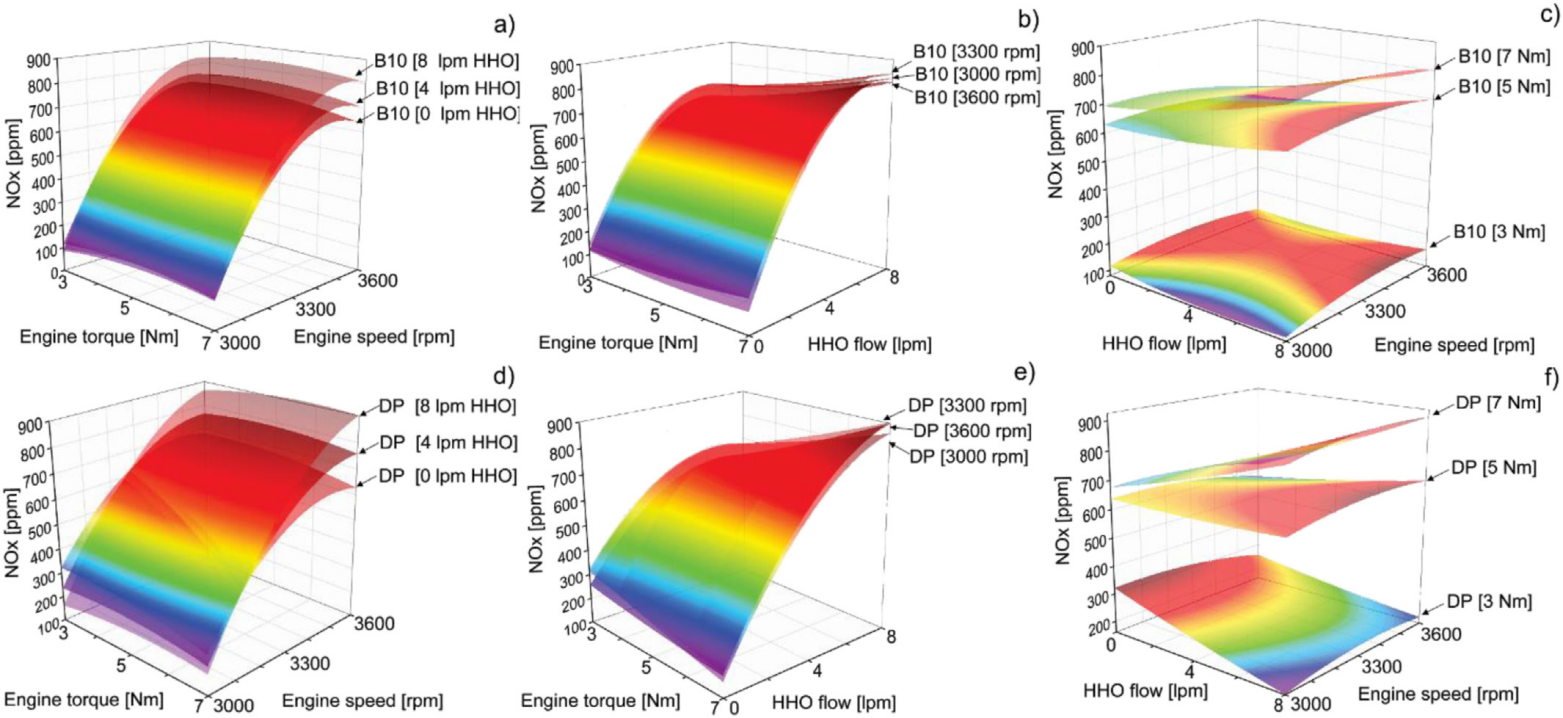
NO_x_ response surfaces of B10 as a function of a) hydrogen volumetric flow, b) engine speed and c) torque and NO_x_ response surfaces of DP as a function of d) hydrogen volumetric flow, e) engine speed and f) torque.

Afterward, Figure 12 shows the response areas for NO_x_ emissions. The results indicate that the minimum values of NO_x_ emissions are presented when operating with low torque and speed and considering a higher hydroxy flow. However, it is important to note that within the behavior of the NO_x_ emissions map, it is observed that, at the lower end of the torque range evaluated, NO_x_ emission constantly increases but then decreases at the upper end. Some studies reported emission maps similar to the results obtained in this study, presenting a similar finding when the torque increases at a constant engine speed [37].

**Figure 13:**
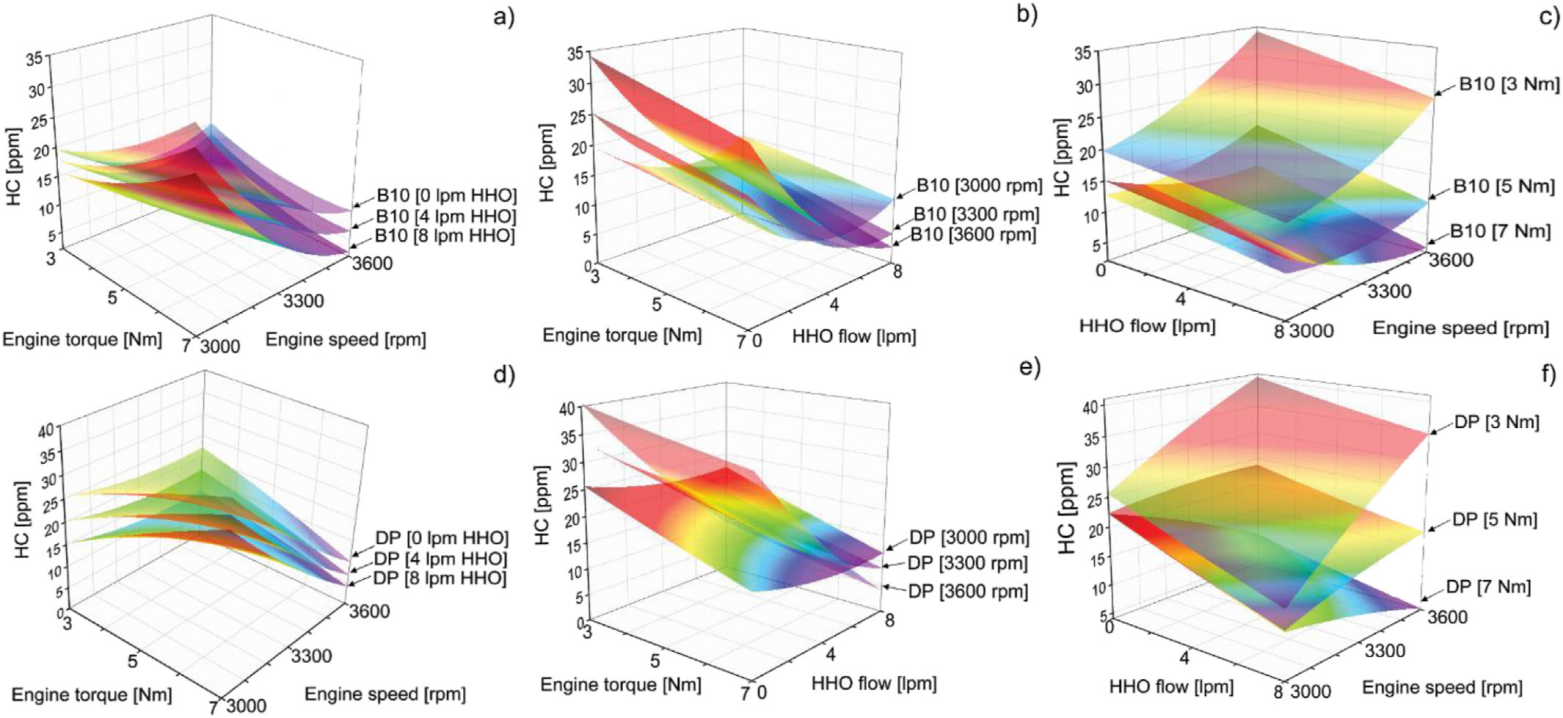
HC response surfaces of B10 as a function of a) hydrogen volumetric flow, b) engine speed and c)torque and HC response surfaces of DP as a function of d) hydrogen volumetric flow, e) engine speed and f) torque.

Lastly, concerning HC emissions, the response surfaces shown in Figure 13 show that the minimum values can be achieved when B10 is selected as the fuel with a hydroxy flow of 8 LPM. In this case, when operating at low and medium load, HC emissions are lower at medium engine speed, while at high load, the engine is required to be operating at high speed as well for lower HC emissions.

The results indicated that hydroxy enrichment in the intake air directly enables high-energy flow to be created throughout the combustion chamber, even before fuel injection, which considerably reduces hydrocarbon formation. The benefit of reducing these polluting emissions is directly linked to the photochemical effect known as smog, a characteristic problem of large cities that takes thousands of lives every day. Another important point of the use of hydroxy that was corroborated in this research is the minimization of CO emissions, which, like unburned hydrocarbons, cause great health problems. In terms of fuel consumption, hydroxy implementation produces a 20% decrease in the SFC, representing an important factor from an economic and environmental viewpoint. It reduces greenhouse gas emissions by burning less coal-based fuel while reducing the required fuel. However, hydroxy enrichment exposed an adverse effect since it promotes greater temperature in the combustion chamber at high loads, thus favoring the NO_x_.

### 3.3 Economic analysis

To study the influence of hydroxy gas on the economy of the engine, the cost of the engine power output $\left(c_{t}\right)$ was calculated, as indicated in Eq. (5).

(5) $$c_{t}=BSFC\times \rho_{f}$$

where BSFC is the brake specific fuel consumption in kg/kWh and $\rho_{f}$ is the price of fuel in USD/kg. Eqs. (6) and (7) were used to determine the price of PD and P [37].

(6) $$\rho_{DP}=\frac{1.11\;USD}{litre}\times \frac{1000\;litre}{m^{3}}\times \frac{1\;m^{3}}{821.5\;kg}=1.35\frac{USD}{kg}$$

(7) $$\rho_{B10}=\frac{0.58\;USD}{litre}\times \frac{1000\;litre}{m^{3}}\times \frac{1\;m^{3}}{827.5\;kg}=0.70\frac{USD}{kg}$$

**Figure 14:**
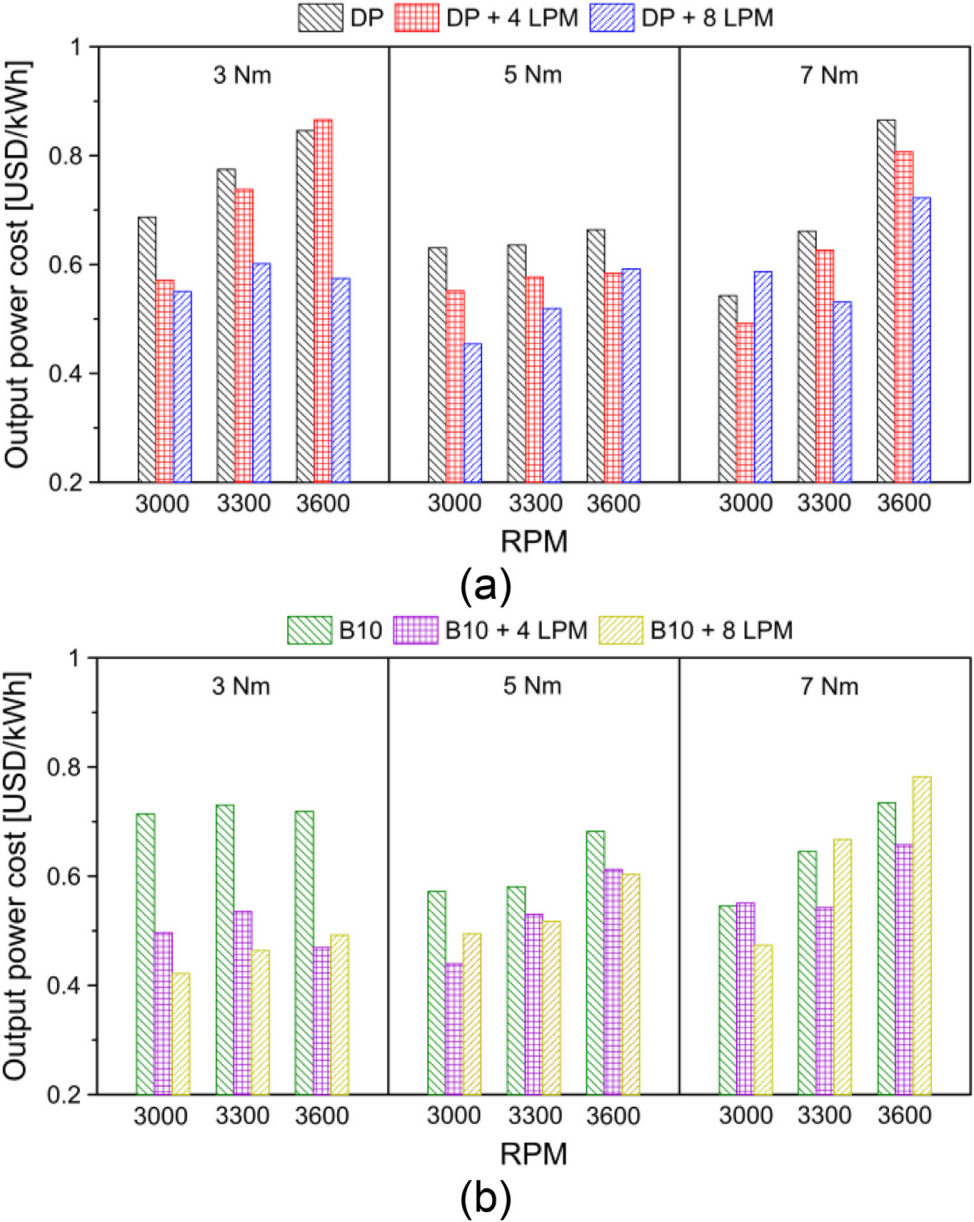
Influence of hydroxy gas on engine power output operating with (a) diesel and (b) biodiesel.

The results of the cost of the engine power output for diesel and biodiesel under the different operating conditions are shown in Figure 14.

From the results of Figure 14, it is possible to show that the addition of hydroxy gas in diesel fuel and biodiesel blend produces a significant reduction in the economic cost of the engine's power output. This is especially important in large-capacity engines due to their high fuel consumption. On average, it was observed that the addition of a flow of 4 LPM and 8 LPM of hydroxy gas in the diesel allows a reduction of 8.0% and 18.4% in the economic cost for each kWh produced by the engine. In the case of the biodiesel blend, the economic cost reduction was 22% and 24%, respectively.

**Figure 15:**
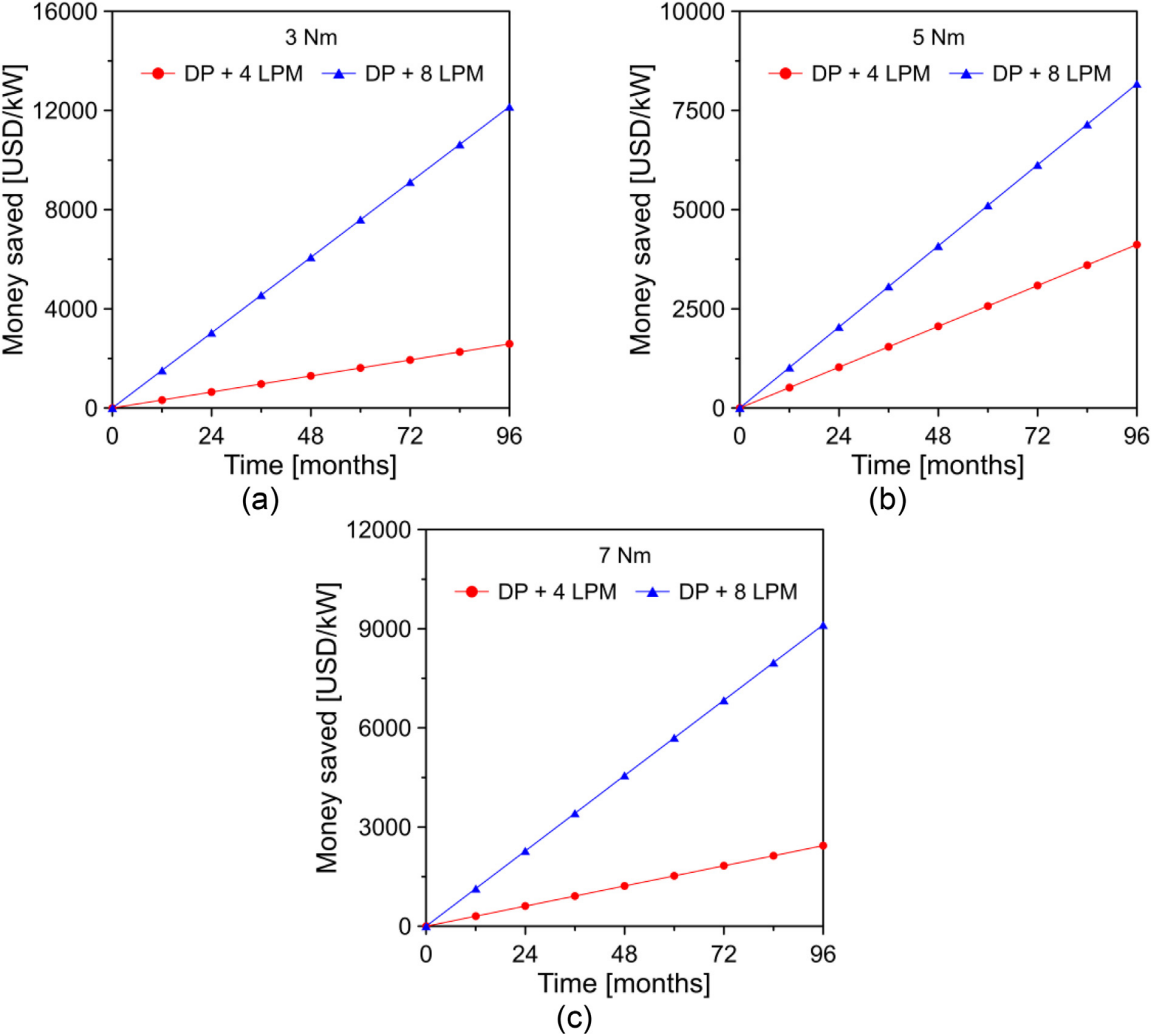
Economic saving of the engine operated with diesel + hydroxy gas for a load of (a) 3 Nm, (b) 5 Nm, and (c) 7 Nm.

**Figure 16:**
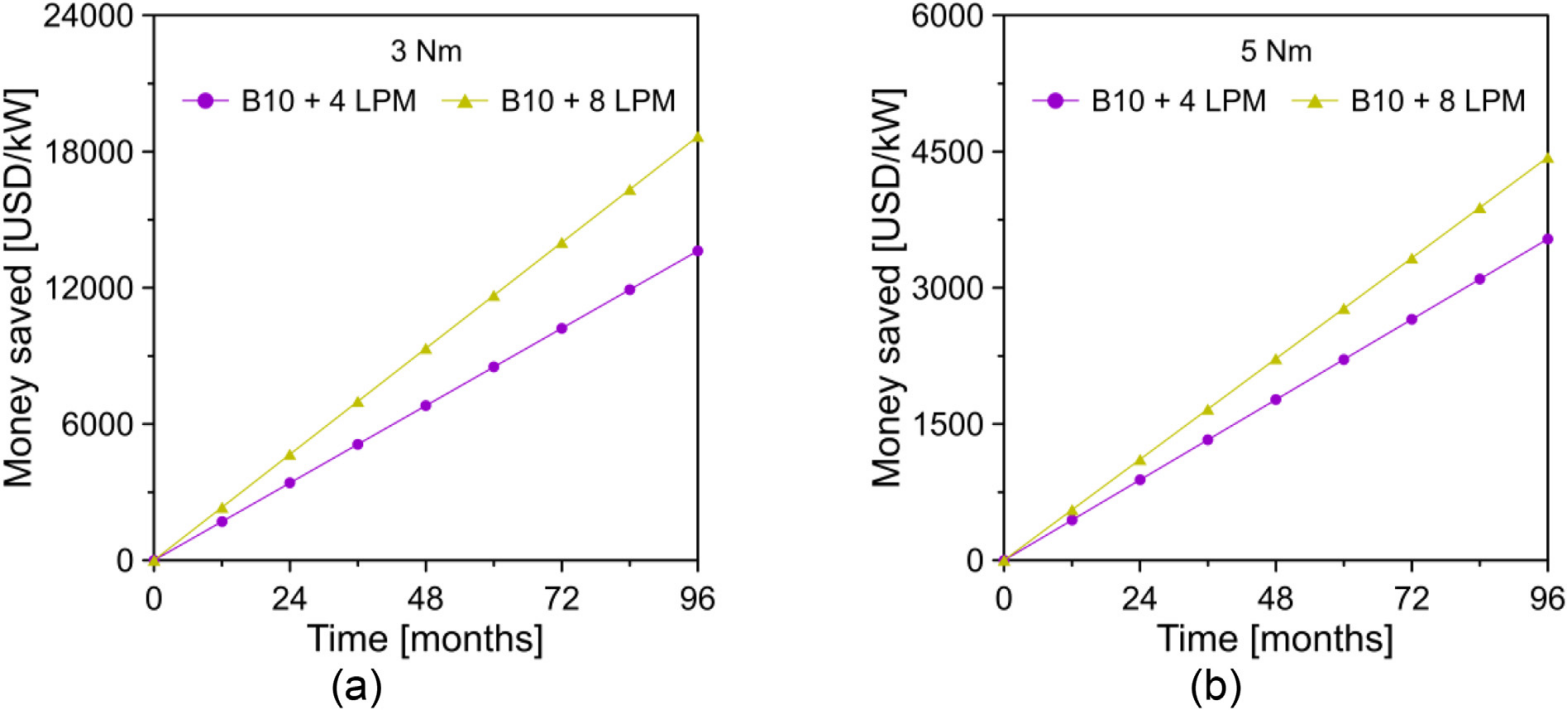
Economic saving of the engine operated with biodiesel + hydroxy gas for a load of (a) 3 Nm and (b) 5 Nm.

The economic saving $\left(c_{s}\right)$ associated with the injection of hydroxy gas in the engine was determined by means of Equation (8) and Equation (9).

(8) $$c_{s,DP+HHO}=\left({BSFC}_{DP}-{BSFC}_{DP+HHO}\right)\times t_{p}$$

(9) $$c_{s,B10+HHO}=\left({{BSFC}_{B10}-BSFC}_{B10+HHO}\right)\times t_{p}$$

where ${BSFC}_{DP+HHO}$ and ${BSFC}_{B10+HHO}$ are the brake specific fuel consumption of the engine operating with hydroxy gas injection. $t_{p}$ is the operating time of the engine, which was defined as 24 h/day, due to it being an engine designed for continuous power generation. The results obtained from the economic savings for diesel and biodiesel are shown in Figures 15 and 16.

From the results of Figures 15 and 16, it was evident that the addition of hydroxy gas is especially beneficial when mixed with commercial diesel. In general, the increase in volumetric flow allows for achieving greater economic savings. However, this effect is not so noticeable when the engine runs on the palm oil biodiesel blend. For an analysis time of 8 years, savings of 4119 USD/kW and 8173 USD/kW were observed with a hydroxy gas injection of 4 LPM and 8 LPM when the engine ran on diesel at a rotation speed of 3300 rpm and a load of 5 Nm. In the case of the engine with the biodiesel mixture, the savings obtained were USD 3538/kW and USD 4439/kW, respectively.

## 4 Conclusions

This study experimentally evaluated the performance of a diesel engine operating in a dual-fuel mode using hydroxy gas as a gaseous fuel. The influence of hydroxy enrichment at a volumetric rate of 4 and 8 LPM was carefully evaluated using PD and B10 as the baseline fuels for the analysis. The incorporation of a complete statistical model to evaluate emissions levels in a multivariate scenario is a unique factor of this work. Moreover, the study incorporates an experimental test bench while characterizing the main subsystems: stationary engine, hydroxy generation module, and DAQ system.

The core findings of the experimental results can be described as follows: for SFC and CO_2_ emissions, PD + 4 LPM of hydroxy reduced these variables on average by 8.6%; PD + 8 LPM of hydroxy, by 20%; B10, by 5%; B10 + 4 LPM of hydroxy, by 22%; and B10 + 8 LPM of Hydroxy, by 21%. At low load, B10 + 8 LPM of hydroxy reduced response variables by 40%; at medium load, PD + 8 LPM of hydroxy by 19%; and at high load, PP + 8 LPM of hydroxy, by 16.5%.

On the other hand, for CO emissions, PD + 4 LPM of hydroxy reduced this variable response on average by 32%; PD + 8 LPM hydroxy, by 48%; B10 by 33%; B10 + 4 LPM of hydroxy by 50%; and B10 + 8 LPM of hydroxy, by 60%. At low load, B10 + 8 LPM of hydroxy reduced the variable response by 40%; at medium load, B10 + 8 LPM of hydroxy by 70%; and at high load, PD + 8 LPM hydroxy, by 69%.

In the case of NO_x_ emissions, PD + 4 LPM of hydroxy reduced this response variable on average by 6.4%; PD + 8 LPM of hydroxy, by 10.5%; B10, by 15%; B10 + 4 LPM of hydroxy, by 23.4%; and B10 + 8 LPM of hydroxy, by 11.6%. At low load, B10 + 8 LPM of hydroxy reduced the variable response by 71%; at medium load, PD + 4 LPM of hydroxy by 5%; and at high load, B10 + 4 LPM of hydroxy, by 0.7%.

Lastly, for the HC emissions, PD + 4 LPM of hydroxy reduced this variable response on average by 23%; PD + 8 LPM of hydroxy by 42%; B10 by 23%; B10 + 4 LPM of hydroxy by 47%; and B10 + 8 LPM of hydroxy, by 58%. At low, medium, and high loads, B10 + 8 LPM of hydroxy showed the best results.

In general, it was demonstrated that hydroxy gas is an alternative gaseous fuel that allows significant savings in internal combustion engines operating with commercial diesel or biodiesel blends. This is especially important in high-capacity engines since the high power output and fuel consumption.

In conclusion, the use of hydroxy as a supplemental fuel proved to enhance the performance of the combustion process, which facilitates the minimization of global emissions and ratifies the benefits it generates both at an environmental and economic level. Moreover, the proposed statistical model stands as a robust tool to evaluate and predict emissions levels in diesel engines. The study features a concrete limitation on the biofuel implementation as only B10 was implemented. Therefore, there is a pressing need for further exploration of biofuel replacement grades, namely B20–B60, that will result in a broader spectrum for dual-fuel operation characterization. Additionally, the incorporation of a comparative assessment of different biofuels blends and additives such as algae oil.

## Declarations

### Author contribution statement

Natalia Duarte: Conceived and designed the experiments; Performed the experiments; Analyzed and interpreted the data; Wrote the paper.

Donovan Arango: Performed the experiments; Analyzed and interpreted the data; Contributed reagents, materials, analysis tools or data.

Geanette Polanco; Guillermo Valencia: Analyzed and interpreted the data; Wrote the paper.

Jorge Duarte Forero: Conceived and designed the experiments; Analyzed and interpreted the data; Wrote the paper.

### Funding statement

This research was supported by the 10.13039/100017432Universidad del Atlántico (ING82-CII 2019).

Geanette Polanco was supported by UiT, The Arctic University of Norway.

### Data availability statement

Data included in article/supp. material/referenced in article.

### Declaration of interest’s statement

The authors declare no conflict of interest.

### Additional information

No additional information is available for this paper.
